# Quad Shot: A Short but Effective Schedule for Palliative Radiation for Head and Neck Carcinoma

**DOI:** 10.4103/0973-1075.58460

**Published:** 2009

**Authors:** Sushmita Ghoshal, Santam Chakraborty, Neeru Moudgil, Manreet Kaur, Firuza D Patel

**Affiliations:** Department of Radiotherapy, Post Graduate Institute of Medical Education and Research, Chandigarh, India

**Keywords:** Dose fractionation, Head and neck cancer, Hypofractionated radiotherapy, Palliative therapy, Quad shot

## Abstract

**Background:**

To evaluate a 2-day course of palliative radiation in patients diagnosed to have inoperable or metastatic head and neck carcinoma.

**Aim:**

To evaluate the symptom relief and quality of life in these patients after this short course of radiation.

**Settings and Design:**

A pilot study was conducted in a tertiary care institute in India.

**Materials and Methods:**

Fifteen patients with stage IV B/C disease, KPS 50-70, were inducted after informed consent. External radiation was given in 2 days, two fractions per day, 6 h apart to a total dose of 14 Gy. Washington University quality of life questionnaire (QOL) was used for assessing QOL before and after radiation. Patients who had more than 50% regression of disease received a second course of similar radiation. All patients were followed up for a mean duration of 6 months.

**Statistical Analysis:**

The Wilcoxon signed rank test was used to evaluate the difference between the QOL scores before and after treatment.

**Results and Conclusions:**

Out of these 15 patients, majority (13) were males and the mean age of the patients was 62 years. After the first course, all patients had good symptom relief, improvement in the QOL, and 13 out of 15 had more than 50% objective response. The short duration of the treatment was favored by the outstation patients and their attendants. It may be concluded that this short course of radiation is an effective tool for palliative radiation and merits a larger randomized trial.

## INTRODUCTION

Squamous cell carcinoma of the head and neck region constitute approximately 20% of the total cancer patient population treated at our center, and about 40% of these patients are treated with only palliative intent. Conventionally, such patients are prescribed a dose of 30 Gy/2 weeks/10 fractions for palliation of symptoms.[1] However, recent phase II trials have suggested alternative hypofractionated schedules for palliation where the overall treatment duration is further reduced.[2] The present study was undertaken to assess one such short course of radiation delivered in 2 days.

## MATERIALS AND METHODS

Between April and July 2008, 15 patients with biopsy-proven squamous cell carcinoma of the head and neck region were inducted in this pilot study after proper informed consent. The inclusion criteria were pathological squamous cell carcinomas of the oropharynx or larynx, stage IV B/C (AJCC Staging System) with Karnofsky performance score (KPS) between 50-70.[3] Patients with prior radiation to the head and neck region were excluded. Thirteen male and two female patients were inducted with a mean age of 62 years.

The University of Washington Quality of Life (UW-QOL) Questionnaire version 4.0 was used to assess the subjective symptoms and QOL before and after 3 weeks of the treatment.[4] It has 12 domains and three general questions based on discrete ordinal responses. A score of zero represents worst and 100 the best possible response.

The ‘Quad Shot’ radiation dose schedule was 14 Gy/2 days/4 fractions as described by Corry *et al*.[2] All the patients were treated in Cobalt^60^ teletherapy units and the gross tumor volume (including the primary tumor and involved nodes) with 2 cm margin was irradiated. Two fractions of radiation were given every day with a minimum 6 h gap between the two fractions. The Biologically Equivalent Dose (BED) for one Quad Shot was 18.9 Gy_10_ and 30.38 Gy_3_ for tumor and late reacting tissues (LQED2 15.75 Gy_10_ and 18.19 Gy_3_), respectively. Patients were reviewed 3 weeks later for response and toxicities.

The tumor response was assessed by WHO criteria,[5] while the NCI CTCAE version 3.0 scoring scheme was used for grading mucosal and dermal toxicities.[6] The sum of the length and breadth of the residual tumor mass was measured and compared with the pretreatment size. If the sum had decreased by more than half (50%) of the pretreatment sum, the ‘Quad Shot’ dose was repeated. If the second Quad Shot was to be delivered, then the BED for the two shots together were 37.8 Gy_10_ and 60.76 Gy_3_ for tumor control and late complications, respectively, assuming no repopulation between two fractions.

Patients were kept on regular follow-up till there was evidence of progression of disease. Statistical analysis was done using the SPSS 12.0 software. The difference between the QOL scores was analyzed using the Wilcoxon Signed Ranks test. All tests were two tailed and *P* value < 0.05 was taken as statistically significant.

## RESULTS

After the first ‘Quad Shot’, an objective response of more than 50% was observed in 13 out of 15 patients, and they received a second course of this treatment. This response was seen both in the site of primary disease and in the metastatic nodes. None of these patients showed more than 50% response after the second Quad Shot course. When assessed 6 weeks after completion of radiation, ten patients had partial response (66.67%), two had static disease, and three had progressive disease. The mean time for disease progression was 12 weeks. All patients were followed this disease progression and thus were alive at last follow-up.

Mucositis was observed in 8 out of 15 patients in which 6 were grade one and 2 were grade two. None of the patients had grade three mucositis. Only 7 out of 15 patients had grade one dermatitis.

Before radiation, pain and difficulty in swallowing were the chief complaints in most of the patients. After ‘Quad Shot’, pain score was found to be improved in 10 out of 15 patients and 5 patients had static score. Before treatment, 11 out of 15 patients required narcotic analgesics (step II and III analgesics); post-radiation, this number was reduced to four. The score for swallowing remained static in 10 and improved in 5 patients. Post-treatment, there was significant mood elevation and decreased anxiety in these patients, as shown in Table 1. None of these patients had worsening of pain or dysphagia, which is commonly associated with radiation-induced mucositis. Although there was no significant change in the scores of chewing, speech, shoulder movements, and consistency of saliva, taste scores deteriorated after radiation in 7 out of 15 patients.

**Table 1:** Median score for each quality of life questionnaire domain in the patient population before and after radiation therapy (difference in between the scores was analyzed using the Wilcoxon signed-rank test)

	Median score ± Standard deviation		*P* value
		
	Before radiation therapy	After radiation therapy	
Pain	25 ± 26.16	75 ± 18.58	0.004*
Swallowing	70 ± 26.99	70 ± 25.85	0.041*
Chewing	50 ± 31.99	50 ± 36.18	0.18
Speech	70 ± 35.79	70 ± 37.83	0.053
Shoulder	100 ± 12.42	100 ± 18.07	1.0
Taste	70 ± 42.73	0 ± 40.96	0.017*
Saliva	100 ± 10.53	100 ± 12.42	0.655
Appearance	75 ± 19.97	100 ± 15.99	0.002*
Activity	50 ± 29.07	50 ± 31.99	0.565
Recreation	75 ± 28.03	75 ± 29.07	0.212
Mood	25 ± 30.56	50 ± 26.50	0.015*
Anxiety	30 ± 26.09	70 ± 30.00	0.009*
Physical domain score	87 ± 20.50	89 ± 20.90	0.184
Social domain score	55 ± 16.47	74 ± 17.04	0.007*
HRQOL 7 days	20 ± 24.14	40 ± 24.91	0.000*
Overall QOL	20 ± 12.79	40 ± 16.32	0.002*
Comp. QOL	25 ± 29.58	75 ± 24.02	0.006*

* Statistically significant difference

**Figure 1:**
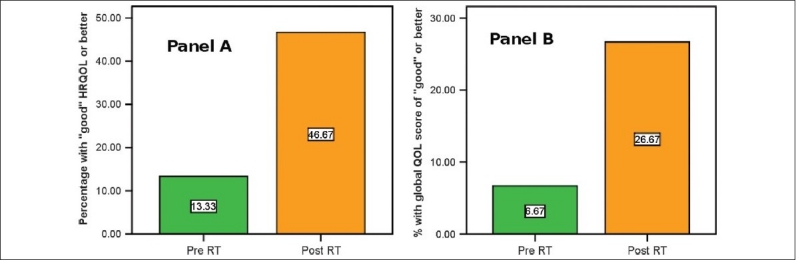
Chart showing the percentage of patients with good or better scores in the HRQOL (Panel A) and overall quality of life questionnaire domains (Panel B) before and after radiation. As can be seen a significantly higher number of patients had a good or better HRQOL and quality of life questionnaire score after treatment

Table 1 presents the median scores (± standard deviation) of the individual domains before and after radiation. A significant deterioration was seen in the taste scores. A significant improvement of scores for all three QOL-related questions was observed when the post-treatment data were compared to the pretreatment values [Figure 1].

## DISCUSSION

The aim of palliative radiation in any advanced cancer is to relieve the symptoms quickly while minimizing the side-effects. In addition, the treatment should be delivered in the shortest possible time considering patients' and caregivers' convenience.

The present study revealed that this 2-day course of radiation was able to produce an objective response in 13 out of 15 patients. The two patients who showed lesser than 50% response were spared any further radiation, while the responders were given a second-cycle ‘Quad Shot’. Unlike Corry *et al.*, we did not give the third cycle as none of our patients showed objective response ≥50% after the second cycle.[2] However, the proportion of patients with reduction or stabilization of disease in our study was comparable to their study (80% and 77%). The mean time for disease progression was 12 weeks in our study, quite similar to the observation of Corry *et al*.[2]

The efficacy of the regime was judged by the symptom relief obtained by the patients and documented by the UW-QOL v4.[4] The treatment effectively palliated pain and dysphagia in the study population, with no evidence of increase in these symptoms after radiation. None of the patients in the present study or in Corry's trial had grade three mucositis, but another hypofractionated radiotherapy trial for similar patients reported 26% grade three mucositis and 11% grade three dermatitis.[7] Similarly in another large Indian study of 505 patients, all patients had patchy mucositis after receiving 20 Gy in five fractions.[8]

During this short period of follow-up, no significant change in salivation was documented but all patients did complain of altered taste. Corry *et al.* reported 20 patients with grade one and two salivary gland toxicity but 16 patients in that study had received the third course of radiation, which may have increased the dose to the salivary glands.[2]

Our study shows a significant improvement in the psychosocial domain, along with the HRQOL and overall QOL, which is of great significance to a patient's being treated with palliative intent. Although almost all physical symptom scores showed improvement, the small numbers made the improvement in the physical domain score insignificant.

The limitations of our trial and similar trials reported in the literature are that they are single armed studies with a relatively short follow-up period, which preclude any comparison with the conventional schedules of radiation. If this regime is found to be as effective as the conventional regimes, it will definitely benefit outstation patients as the duration of stay away from home will be shorter. In addition, the shorter course of radiation would be logistically better for the treating center. A prospective randomized trial is underway to compare ‘Quad Shot’ regime with conventional palliative radiation practiced at our center.
